# Usability and effectiveness of Suprathel^®^ in partial thickness burns in children

**DOI:** 10.1007/s00068-016-0708-z

**Published:** 2016-07-18

**Authors:** Z. M. Rashaan, P. Krijnen, J. H. Allema, A. F. Vloemans, I. B. Schipper, R. S. Breederveld

**Affiliations:** 1 Department of Surgery, Leiden University Medical Center, PO Box 9600, 2333 ZA Leiden, The Netherlands; 2 Burn Centre, Red Cross Hospital Beverwijk, Vondellaan 3, PO Box 1074, 1942 LE Beverwijk, The Netherlands; 3 Department of Surgery, Haga Teaching Hospital-Juliana Children’s Hospital, PO Box 60605, 2506 LP The Hague, The Netherlands; 4 Associations of Dutch Burn Centers, PO Box 9100, 3079 DZ Beverwijk, The Netherlands

**Keywords:** Suprathel^®^, Partial thickness burns, Children, Usability, Effectiveness

## Abstract

**Purpose:**

Evaluation of usability and effectiveness of Suprathel^®^ in the treatment of partial thickness burns in children.

**Methods:**

A prospective, observational study to evaluate adherence of Suprathel^®^ to the wound bed, reepithelialization time, grafting, wound colonization and infection, pain, dressing changes, length of hospital stay (LOS) and scar formation.

**Results:**

Twenty-one children (median age 2.4 years, range 5 months–14 years) with a median total body surface area (TBSA) of 4 % (range 1–18) were included. Median LOS was 10 days (range 3–20). Median outer layer dressing changes was 3 (range 1–14). Suprathel^®^ was only adherent in wounds debrided with Versajet^®^. Median reepithelialization time was 13 days (range 7–29). Three patients needed a split skin graft. There were 7 (33 %) patients with wound colonization before application of Suprathel^®^. This increased to 12 (57 %) patients during treatment. One patient developed a wound infection. Median visual analog scale (VAS) scores for background and procedural pain in patients >7 years were 3.2 (range 2–5) and 3.5 (range 2–5), respectively. In younger patients, median background and procedural COMFORT-B scores were 13.8 (range 10–23) and 14.8 (range 13–23, *p* = 0.03), respectively. Patient and Observer Scar Assessment Scale (POSAS) scores were favorable after 3 and 6 months post burn.

**Conclusions:**

Suprathel^®^ provides potential advantages regarding pain and scar formation, but extensive wound debridement is needed to achieve adequate adherence.

## Introduction

Although partial thickness burns are the most common burn injuries among children, there is no ‘gold standard’ for the optimal treatment of this type of burn injury [1, 2]. The treatment of partial thickness burns focuses on undisturbed wound healing by providing a moist wound environment, removal of exudate, prevention of infection, and minimization of pain, scar formation and functional impairment [1, 3, 4].

In the last few decades, significant progress has been made in the field of (semi)synthetic wound dressings to meet the above requirements. One of the latest innovations in this field is the development of Suprathel^®^. Suprathel^®^ (PolyMedics Innovations GmbH, Filderstadt, Germany) is a biosynthetic, non-animal derived wound dressing that imitates the protective properties of the human epithelium by adhering to the wound bed at body temperature [5, 6]. The microporous membrane of Suprathel^®^, which has an elongation capacity of up to 250 %, is water-soluble and composed of a co-polymer (terpolymer) of poly-dl-lactide, trimethylene carbonate and ε-caprolactone [5, 7]. The porous property of Suprathel^®^ is intended to prevent accumulation of wound exudate and thereby preventing also wound infection. Also, a moist wound environment is supposed to be established, which may contribute to an optimal wound healing. Suprathel^®^ is transparent after application to the wound bed which enables inspection of the wound without removing the dressing [8].

Literature on effectiveness of Suprathel^®^ in pediatric burn patients is scarce. Only two recent non-comparative studies reported good results in terms of wound healing in children with partial thickness burns [6, 9]. However, no studies reported validated data on the wound colonization, scar formation and pain after application of Suprathel^®^ on the wound and before and after each outer layer dressing changes.

This study evaluates the usability and effectiveness of Suprathel^®^ in the treatment of partial thickness burns in children.

## Patients and methods

This observational, prospective study was conducted in the Juliana Children’s Hospital, The Hague and in the Beverwijk Burn Centre of the Red Cross Hospital, Beverwijk, The Netherlands.

### Patients

Between November 2011 and January 2013, all consecutive patients younger than 18 years with partial thickness burns who were seen in these hospitals within 48 h after injury, were eligible for this study. Patients were excluded if they had only facial burns, if they previously had been treated elsewhere for their burn wounds or if they were expected to be non-compliant with their treatment, for example because of a profound language barrier.

### Treatment protocol

All included patients underwent the same treatment protocol. Suprathel^®^ was applied to the wound after administrating oral analgesics in the outpatient department or under general anesthesia (propofol, fentanyl) in the operating theatre within 48 h after injury. The burn wounds were cleaned by rinsing and superficial debridement of loose skin remnants and blisters or by using a Versajet^®^ hydrosurgery system for surgical wound debridement [10]. Thereafter, a Suprathel^®^ film was cut to adequate dimensions to cover the complete burned area, whereupon a multilayer Vaseline gauze dressing was applied to keep the Suprathel^®^ separated from the outer absorbing dressings. Depending on the extension of the burns, patients were then either admitted to the ward or discharged and regularly seen in the outpatient clinic until complete wound healing. Thereafter, patients were seen at 3 and 6 months after injury. Suprathel^®^ was left in situ until 95 % reepithelialization had been achieved, while the outer dressings were changed routinely every 3–5 days. During these dressing changes, only outer layer dressings were removed, adherent Suprathel^®^ was left untouched, and loose Suprathel^®^ over the healed area was trimmed. If the Suprathel^®^ was completely detached from the unhealed wound bed, it was removed after which the exposed wound was treated with a topical agent. At the 10th–14th day post burn, it was decided whether a skin graft was needed. Reasons for grafting were expected absence of progressive wound healing in the next 7–11 days and full thickness burns.

Swabs for semi-quantitative analysis of wound microbial flora were taken on admission, before the application of Suprathel^®^ and during each outer layer dressing change. Wound colonization was defined as at least one positive bacterial culture from the wound [11]. Infection was defined as a combination of skin redness, pain, swelling, tenderness, warmth, fever or pus draining from the skin with a positive wound colonization [12]. If infection occurred, Suprathel^®^ was removed from the wound. Based on the outcome of the swabs, infected wounds were treated with an appropriate local antiseptic.

### Data

Baseline characteristics were recorded including gender, age, cause and location of the burn, depth of the burns (superficial or deep partial thickness), time from burn to start of treatment in days, and percentage of affected total body surface area (TBSA). Superficial partial thickness burns (SPTB) were clinically defined at the acute stage as painful burn wounds that had a moist pink colored appearance, with intact or disrupted blisters and with a capillary refill within less than seconds. Deep partial thickness burns (DPTB) were defined as painful wounds with a dry and red-to-pale appearance with pale blotchy patches, with intact or disrupted blisters and a capillary refill after more than 3 s [13].

Usability of Suprathel^®^ was evaluated by its adherence to the wound bed and the number of (outer layer) dressing changes. Effectiveness of Suprathel^®^ was evaluated in terms of reepithelialization time, need for skin grafting, wound colonization and infection, pain, length of hospital stay (LOS) and scar formation.

Reepithelialization time was defined as the number of days until at least 95 % reepithelialization of the wound, judged by an experienced burn surgeon. The number of burn wounds that were treated with Suprathel^®^ and required secondary (surgical) intervention were also determined. Pain was measured before each outer layer dressing change to measure background pain and after each outer layer dressing change to evaluate procedural pain. Patients older than 7 years scored pain on a visual analog scale (VAS), a continuous horizontal 10-cm line ranging from 0 (no pain) to 10 (worst possible pain). The COMFORT-Behavior scale was scored by trained pediatric nurses to measure pain in younger patients. This COMFORT-B scale contains six behavioral items including alertness, calmness, respiratory response or crying, muscle tone, physical movement, and facial tension. For each item, the response categories range from 1 (‘no distress’) to 5 (severe distress) leading to an overall score ranging from of 6 to 30 [14].

Scar formation was assessed at 3 and 6 months post burn, using the Patient and Observer Scar Assessment Score (POSAS [15, 16]). The POSAS consists of an observer scale, which is scored by an experienced burn specialist, and a patient scale, which is scored by the patient. The observer scale includes items on vascularization, pigmentation, thickness, relief and pliability, while the patient scale measures pain, itching, color, stiffness, thickness and irregularity of the scar. The items on both scales are scored on a 10-point scale, ranging from 1 (‘normal skin’) to 10 (‘worst imaginable scar’). Patients above 13 years of age score the patient scale themselves, whereas parents or caregivers fulfill this task for younger patients.

### Statistical analysis

For this observational study, no formal sample size calculation was performed. A sample size of about twenty patients was considered sufficient to obtain insight into the usability and effectiveness of Suprathel^®^ during the inclusion period. Data were stored in an SPSS database version 17 (SPSS Inc., Chicago IL) and described using summary statistics (median/range or number). Categorical data were compared between the groups using Fisher’s exact test. Comparison of the time to reepithelialization between SPTB and DPTB or wounds with/without bacterial colonization was performed using the log-rank test. Pain scores before and during application of Suprathel^®^ on the wound bed were compared within patients using the Wilcoxon signed-rank test for paired data. Two-sided *p* values <0.05 were considered as statistically significant.

## Results

### Patients

Twenty-one patients (10 male, 11 female) with a median age of 2.4 years (range 5 months–14 years) were treated with Suprathel^®^ during the inclusion period (Table 1). All patients were in good general health without comorbidities. Most burns were caused by scalding (*n* = 19) and affected the anterior trunk (*n* = 10) or the extremities (*n* = 17). Eleven (52 %) patients were treated in an outpatient setting, and ten (48 %) patients were admitted to hospital. Median TBSA at admission was 4 % (range 1.0–18.0). Median TBSA of the patients that were treated in outpatient settings was 2.5 % (range 1.0–5.0), while median TBSA of admitted patients was 6.0 % (range 3.5–18.0). At the initial assessment, the burns of twelve (57 %) patients were classified as SPTB and as DPTB in nine (43 %) patients. Median LOS of the admitted patients was 10 days (range 3–20).

**Table 1:** Patient characteristics of 21 children with partial thickness burns

Age, median (range)	2.4 years (5 months–14 years)
Gender, *n* (%)	
Male	10 (48)
Female	11 (52)
Burn cause, *n* (%)	
Scald	19 (90)
Flash	1 (5)
Flame	1 (5)
Location of burn, *n* (%)	
Head and neck	2 (7)
Trunk (anterior)	10 (33)
Trunk (posterior)	1 (3)
Upper extremities	9 (30)
Lower extremities	8 (27)
Treatment, *n* (%)	
In outpatient clinic	11/21 (52)
Admitted	10/21 (48)
% TBSA, median (range)	
In patients treated in outpatient clinic	2.5 (1.0–5.0)
In admitted patients	6.0 (3.5–18.0)
Total	4.0 (1.0–18.0)
Depth of burn, *n* (%)	
SPTB	12 (57)
DPTB	9 (43)

*TBSA* total body surface area, *SPTB* superficial partial thickness burns, *DPTB* deep partial thickness burns

### Usability of Suprathel^®^

#### Adherence to wound bed

In most patients with SPTB (11/12, 92 %), a superficial wound debridement was performed, while the wounds of patients with DPTB were mostly debrided by Versajet^®^ hydrosurgery (7/9, 78 %) (Table 2). The median time to application of Suprathel^®^ on the wound was 1 day (range 0–2) post burn. In nine (43 %) patients, Suprathel^®^ was completely detached from the wound surface at the first outer layer dressing change (Table 2). All cases of complete detachment occurred in the group in which only superficial wound debridement had been performed (9/13, 69 %). In contrast, no detachment of Suprathel^®^ from the wound was seen in the group in which debridement had been performed by Versajet^®^ hydrosurgery (0/8, *p* = 0.005).

**Table 2:** Measures of usability and effectiveness of Suprathel^®^ in 21 children with partial thickness burns

Debridement, *n* (%)	
SPTB	
Superficial debridement	11/12 (92)
Versajet^®^ hydrosurgery	1/12 (8)
DPTB	
Superficial debridement	2/9 (22)
Versajet^®^ hydrosurgery	7/9 (78)
Time until application of Suprathel^®^ in PBD, median (range)	1 (0–2)
TBSA of wound area treated with Suprathel^®^, median (range)	4 (1–18)
Adherence till wound healing, *n* (%)	
Yes	12 (57)
No	9 (43)
Number of dressing changes, median (range)	
During Suprathel^®^ adherence	3 (1–14)
After detachment of Suprathel^®^	2 (0–7)
Time to reepithelialization in days, median (range)	
Total	13 (7–29)
Wound without bacterial colonization	13 (7–18)
Wound with bacterial colonization	15 (9–29)
Split skin graft, *n* (%)	3 (14)
Length of hospital stay, median (range)	10 (3–20)

*SPTB* superficial partial thickness burns, *DPTB* deep partial thickness burns, *PBD* post burn day, *TBSA* total body surface area

#### Dressing changes

The median number of outer layer dressing changes was three (range 1–14) in patients in whom Suprathel^®^ was adherent. However, in nine (43 %) patients in whom Suprathel^®^ did not adhere to the wound bed, another dressing was applied. The median number of these dressing changes was two (range 0–7) (Table 2).

### Effectiveness of Suprathel^®^

#### Reepithelialization time and need for skin grafting

The median reepithelialization time was 13 days (range 7–29). No significant difference in time to reepithelialization was found between SPTB and DPTB, 11 days (range 7–29) and 15 days (12–19; *p* = 0.26), respectively. The median time to reepithelialization was 15 days (range 9–29) for wounds with bacterial colonization, and 13 days (range 7–18) for non-colonized wounds (*p* = 0.45). One patient, who suffered a wound infection, healed in 29 days. All SPTB healed without surgical intervention, whereas three of the patients with DPTB needed a split skin graft.

#### Colonization and infection

Seven (33 %) patients showed wound colonization before application of Suprathel^®^ to the wound. During treatment with Suprathel^®^, the number of patients with wound colonization increased to 12 (57 %). Various microorganisms were found in the colonized wounds: *Staphylococcus aureus*, *Pseudomonas* and *group B streptococcus*, and *Acinetobacter baumannii*. One patient in the DPTB group showed signs of infection with *S. aureus*.

#### Pain

Patients younger than 7 years had a median background COMFORT-B score of 13.8 (range 10–23), while their median procedural score was 14.8 (range 13–23, *p* = 0.03). There was no difference between pain scores given by the older patients before (median 3.5, range 2–5) and during (median 3.2, range 2–5) outer layer dressing changes (*p* = 1).

#### Scar formation

Table 3 presents the median POSAS scores at 3 and 6 months post burn. Most of the POSAS scores by observers and patients/parents were mainly in the lower third of the range, reflecting a good scar quality after 6 months post burn.

**Table 3:** Scores on the Patient and Observer Scar Assessment Score (POSAS) for evaluation of scar formation in 21 children with partial thickness burns treated with Suprathel^®^

	3 months post burn	6 months post burn
**Observer**		
Vascular, median (range)	3 (2–6)	2.5 (1–7)
Pigmentation, median (range)	3 (2–7)	2.5 (2–8)
Thickness, median (range)	3 (1–6)	2.5 (1–4)
Relief, median (range)	2 (1–6)	2.5 (1–4)
Pliability, median (range)	2 (1–8)	2 (1–5)
Surface, median (range)	2 (1–7)	2 (1–3)
Overall opinion, median (range)	3 (1–7)	2.5 (1–5)
**Patient/parents**		
Pain, median (range)	1 (1–8)	1 (1–2)
Itching, median (range)	3 (1–8)	2.5 (1–5)
Color, median (range)	6 (3–8)	6 (2–9)
Pliability, median (range)	2 (1–6)	2.5 (1–8)
Thickness, median (range)	2 (1–8)	3 (1–8)
Irregularity, median (range)	2 (1–9)	3 (1–8)
Overall opinion, median (range)	4.5 (2–9)	3.5 (1–7)

## Discussion

Suprathel^®^ is a potentially good alternative for biological wound dressing, because it is not derived from animals and, therefore, acceptable for all patient groups. To the best of our knowledge, this study is the only detailed prospective study including long-term results on the usability and effectiveness of Suprathel^®^ in the treatment of partial thickness burns in children. The data resulting from this study, which were obtained by validated measurement tools when possible, provide more insight into the usability and effectiveness of this treatment in daily practice and can be used to design future comparative studies with other types of dressings in the treatment of partial thickness burns in children.

The lack of adherence of Suprathel^®^ to the wound bed seems to be attributable to the extent of debridement technique. Adherence of Suprathel^®^ was achieved when the Versajet^®^ system had been used, while no adherence was seen in most cases when superficial debridement had been performed. Three studies reported on the adherence of Suprathel^®^ to the wound bed in the treatment of partial thickness wounds and found excellent material adherence [6, 7, 9]. However, the effect of the extent of wound debridement on the adherence is not clear in these studies, because either different debridement techniques were used or no information was reported on the debridement technique. Our study suggests that extensive wound bed debridement might be a requirement for adequate adherence of Suprathel^®^ to the wound.

The results regarding time to reepithelialization in our study were comparable to those of a previous non-comparative study on the treatment of partial thickness burns in children with Suprathel^®^ [6, 9]. In two recent systematic reviews on the treatment of partial thickness burns in children, the mean time to reepithelialization in randomized controlled trials (RCT) that used other (semi)synthetic dressings varied between 7.5 and 23.6 days [1, 17]. In adults, no difference in reepithelialization was found when Suprathel^®^ was compared to other (semi)synthetic dressings (Biobrane^®^ [18] and Omiderm^®^) [19] or split-thickness skin graft (STSG) [8] in the treatment of partial thickness burns. In pediatric patients, a short reepithelialization time is important as other studies have shown a low risk of developing hypertrophic scars and contractures in burn wounds that healed within 21 days [20, 21]. Our study seems to confirm this finding as healing of the burn wound in one patient took more than 21 days, due to wound infection, after which this patient developed a hypertrophic scar and had the worst POSAS score in our study.

Three children (14 %) in our study received a skin graft, because no spontaneous wound healing was expected within 21 days after the burn injury. In other studies, the need for skin grafting varied between 0 and 17 % in children with partial thickness burns that were treated with other (semi)synthetic dressings than Suprathel^®^ [22–25]. However, comparing these results with the current study should be done with caution, because in these studies, no indication for and timing of skin grafting were reported. In our hospital, the standard care for the partial thickness burns is aimed to achieve reepithelialization, with or without skin grafting, within 21 days. The aims of relatively early skin grafting are to allow the superficial area to heal, to reduce the risk of infection and inflammatory syndrome and to improve the functional result by minimizing the risk of scar formation [26]. This approach may have led to a relatively high number of skin grafts in our study.

Scar formation is one of the most important outcomes that is evaluated rarely in children with partial thickness burns [1]. Adequate follow-up and evaluation of the scars with validated measurement tools are vital to manage scar formation. Cubison et al. demonstrated that hypertrophic scars had developed four months post burn in children with a TBSA of more than 5 % [27]. Therefore, we evaluated our patients at 3 and 6 months post burn. To our knowledge, no previous study evaluated the scar formation with a validated method in pediatric patients with partial thickness burns that were treated with Suprathel^®^. In adults, treatment with Suprathel^®^ has shown a better scar quality compared to STSG in the treatment of partial thickness burns after 90 days post burn [8]. On the other hand, two other studies have shown no difference in hypertrophic scar formation when Suprathel^®^ was compared to Omiderm^®^ or Biobrane^®^ in the treatment of partial thickness burns in adults [18, 19]. We found favorable scar quality in our study after 6 months post burn according to the POSAS scores.

Suprathel^®^ forms a surrogate, natural barrier for microorganisms, that is intended to prevent accumulation of wound exudate and contains polylactic acid which reduces the local wound pH [28]. These properties may theoretically minimize the risk of wound colonization and wound infection and may, therefore, support optimal reepithelialization. However, an in vitro study by Ryssel et al. showed insufficient evidence for an antiseptic effect of Suprathel^®^ [28]. Our study seems to support these results since the number of patients with a colonized wound did not decrease after application of Suprathel^®^. Nevertheless, the current study found no apparent difference in reepithelialization between wounds with and without bacterial colonization. The role of microorganisms in delayed wound healing is not clearly established [29]. Some studies found that the concentration of microorganisms is an important determinant for wound healing process [30–32], while other studies found the role of microorganisms less important in delayed wound healing [33–35]. On the contrary, the only patient with wound infection in our study had delayed reepithelialization. In the literature, the association of burn wound infection and delayed reepithelialization is well established [36, 37].

This study found minimal changes between background pain and procedural pain. There was a statistically significant increase in COMFORT-B scores in the youngest children, but the difference in scores was minimal and may not be clinically relevant. Everett et al. also found minimal pain levels after application of Suprathel^®^ on the wound in the treatment of partial thickness burns in children [9]. An explanation for these minimal changes between background pain and procedural pain might be that no manipulation of the wound bed occurs when Suprathel^®^ adheres to the wound bed. Manipulation of the wound bed is the main cause of the procedural pain which is the most intense pain in burn patients [38, 39]. Inadequate management of burn injury pain increases patients’ anxiety for the dressing changes, reduces the effectiveness of analgesia and, in the long-term, changes pain perception and related behaviors [40–42]. Thus, novel burn treatments focus on reducing burn injury pain, for instance, by reducing the number of dressing changes. One study described analgesic response of the outer layer dressing changes as “very good” to “excellent” in children with partial thickness burns that were treated with Suprathel^®^. However, pain was not scored with a validated measurement tool in this study [6]. Studies in adult patients have shown lower pain scores for patients that were treated with Suprathel^®^ compared to Omiderm^®^ and Mepilex^®^ in the treatment of partial thickness burns and donor sites of skin grafts, respectively [7, 43].

Since Suprathel^®^ is porous and permeable to fluid, it requires an outer layer absorbing dressing to absorb the extensive amount of wound exudate. The number of outer layer dressing changes is not previously described in studies in patients with partial thickness burns that were treated with Suprathel^®^. The number of outer layer dressing changes in our study is comparable with the mean number of dressing changes between 1.5 and 7.5 in RCTs that used (semi)synthetics dressings in the treatment of partial thickness burns in children [23, 24, 44, 45].

A limitation of this study is the small sample size so that the power of the study was too low to detect clinically relevant differences between subgroups of patients. Another limitation of this study is that the burn depth was evaluated only by clinical assessment. It has been demonstrated that the combination of the clinical assessment and laser Doppler imaging (LDI), that evaluates the difference in perfusion of the microvascular blood flow of the wound, is more accurate and reliable way to evaluate the burn depth than clinical evaluation only [46]. Finally, no comparison with other (semi) synthetic wound dressing is performed due to the non-comparative nature of this study.

## Conclusions

Our study on the usability and effectiveness of Suprathel^®^ in the treatment of partial thickness burns in pediatric patients found potential advantages of Suprathel^®^ treatment regarding pain and scar formation as compared to published results on (semi)synthetic dressings in the literature. No clear advantages were found regarding reepithelialization, need for grafting, wound colonization and infection and dressing changes. Also, extensive wound debridement is needed to achieve adequate Suprathel^®^ adherence. Randomized controlled trials are needed to evaluate the efficacy of Suprathel^®^ compared to other (semi)synthetic dressings in the treatment of partial thickness burns in pediatric patients.
